# History of the medical education accreditation system in Korea: implementation and activities in the early stages

**DOI:** 10.3352/jeehp.2020.17.29

**Published:** 2020-10-21

**Authors:** Kwang-ho Meng

**Affiliations:** Department of Preventive Medicine, College of Medicine, The Catholic University of Korea, Seoul, Korea; Hallym University, Korea

**Keywords:** Academies and institutes, Accreditation, Medical education, Medical schools, Republic of Korea

## Abstract

Following the opening of 12 new medical schools in Korea in the 1980s, standardization and accreditation of medical schools came to the forefront in the early 1990s. To address the medical community’s concerns about the quality of medical education, the Korean Council for University Education and Ministry of Education conducted a compulsory medical school evaluation in 1996 to see whether medical schools were meeting academic standards or not. This evaluation was, however, a norm-referenced assessment, rather than a criterion-referenced assessment. As a result, the Accreditation Board for Medical Education in Korea (ABMEK) was founded in 1998 as a voluntary organization by the medical community. With full support of the Korean medical community, ABMEK completed its 1st cycle of evaluations of all 41 medical schools from 2000 to 2004. In 2004, ABMEK changed its name to the Korean Institute of Medical Education and Evaluation (KIMEE) as a corporate body. After that, the Korean government paid closer attention to its voluntary accreditation activities. In 2014, the Ministry of Education officially recognized the KIMEE as the 1st professional institute for higher education evaluation and accreditation. The most important lesson learned from ABMEK/KIMEE is the importance of collaboration among all medical education-related organizations, including the Korean Medical Association.


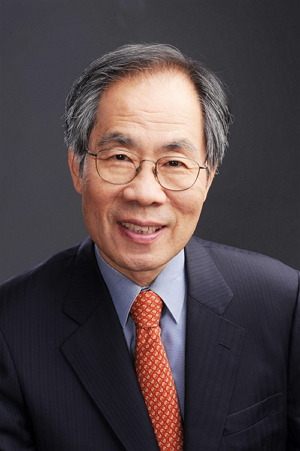


## Introduction

### Background/rationale

On November 8, 2019, the 20th-anniversary symposium of the Korean Institute of Medical Education and Evaluation (KIMEE) was held at the Seoul Press Center International Conference Room. The symposium’s main topic was “Past, present, and future of the Korea Medical Education Evaluation Institute” [1]. It is worthwhile to revisit the history of medical education accreditation activities conducted by the Accreditation Board of Medical Education and Evaluation of Korea (ABMEK) and the KIMEE starting in the late 1990s. ABMEK was launched in July 1998, and its succeeding organization, KIMEE, was established on November 21, 2003. On February 27, 2004, the KIMEE became an independent corporation through registration with the Ministry of Health and Welfare. On May 12, 2014, the KIMEE was designated as a higher education program evaluation and accreditation institute by the Ministry of Education.

On September 19, 2016, KIMEE became the 5th agency recognized by the World Federation for Medical Education (WFME) for the accreditation of medical education. It was the 1st agency to be recognized by the WFME in Asia [2] (Table 1).

Another remarkable achievement of the KIMEE was reflected in the revision of the Medical Service Act. Specifically, in February 2012, the Ministry of Health and Welfare revised the Medical Service Act to state that graduates from medical schools that have not been accredited by the KIMEE cannot take the Korean Medical Licensing Examination starting in 2017, as follows:

Article 5 (Licenses for Medical Doctors, Dentists or Oriental Medical Doctors) (1) A person who intends to become a medical doctor, dentist, or oriental medical doctor shall meet any of the following qualifications and be licensed by the Minister of Health and Welfare after passing the relevant national examination prescribed in Article 9. (Amended by Act No. 9932, January 18, 2010; Act No. 11252, February 1, 2012)
1. A bachelor’s degree holder who has graduated from a university or college, with a major in medical science, dentistry, or oriental medical science, which has obtained accreditation from an accredited institution referred to in Article 11-2 of the Higher Education Act (hereinafter referred to as “evaluation and certification body”; and the accreditation there from shall be referred to as “accreditation from the evaluation and certification body”)
2. A master’s or a doctor’s degree holder who has graduated from a professional graduate school, such as a medical school, dental school, or oriental medical school which has obtained accreditation from the evaluation and certification body

Another innovative step was the revision of part of the Enforcement Decree of the Higher Education Act to mandate the accreditation of all medical schools by June 2016. The medical school accreditation project, which was started voluntarily despite a challenging environment, was recognized by the Korean government’s legal and institutional support. Evidence of the impact of these legal changes is furnished by the fact that the Seonam University Medical School, which failed accreditation in 2013 and 2016 by the KIMEE, was eventually closed in February 2018.

### Objectives

This paper describes the history of the medical education accreditation system in Korea before 2004. The history of the early stages of medical education accreditation has been summarized in two earlier publications [3,4]. However, these two publications are not comprehensive, but rather contain the minutes of meeting records or descriptions of institutional relationships with the KIMEE. Another purpose of this paper is to provide a supplemental literature source for researchers interested in Korea’s medical education accreditation system.

## Historical background of the introduction of the medical education accreditation system until 2004

The accreditation system includes an “evaluation of medical schools in terms of their objectives and emphasis on the accreditation process as a consultation rather than an inspection” by outside experts [5]. Therefore, the accreditation of a medical school can furnish proof of a medical school’s educational environment and educational programs to satisfy the standards of the professional accreditation body for appropriately nurturing the physicians needed by society.

In Korea, a simple assessment of the status of medical education at medical schools has been conducted since 1979. The booklet “Status of medical school education,” published by the Korean Association of Medical Colleges (KAMC), is a prime example. Through this collection of information on the status of medical education, administrators of each university can identify relative deficiencies or areas for improvement by comparing its curriculum and educational environment with those of other universities. However, this booklet alone was insufficient as a source to determine whether a medical school’s educational level is sufficient from the perspective of social accountability. Instead, it was necessary to introduce an accreditation system in which an independent organization would assess whether a medical school’s curriculum and educational environment fit particular item-level criteria.

Issues across medical education, including medical school accreditation, have been actively discussed in medical education seminars at the medical school dean’s conference or dean’s council since the 1970s. As many medical schools were established in the 1970s and 1980s, the quality of medical education became a major concern in the medical field [4]. However, the main background of the introduction of Korea’s medical school accreditation system was not merely to avert the establishment of numerous new medical schools. Although medical school accreditation served to prevent an excessive number of medical schools from being established, accreditation standards for existing medical schools are a distinct issue from the standards necessary for new medical schools. Medical school deans have discussed how to improve the overall level of medical education in Korea since the 1970s. Examples of medical education reform for the 21st-century generation in the United States, the United Kingdom, and Australia since the 1980s also functioned as models for the introduction of an accreditation system in Korea [6,7].

It is clear that at this time, the Ministry of Education was also interested in medical education accreditation in any form to improve the medical education and the educational environment at medical schools in Korea. In 1981, the Ministry of Education published a research report on premedical, medical, nursing, dental, and oriental medicine education programs in universities [8]. This evaluation consisted of a university’s self-assessment report and a local visit evaluation by professors from other universities. Each department’s educational contents and educational environment levels were classified as A, B, and C. In 1993, the Korean Council for University Education commissioned a group of medical educators to conduct an evaluation study of 23 medical schools [9]. This evaluation was also not sufficiently comprehensive to serve as an “accreditation” for medical school education programs.

**Fig. 1.**
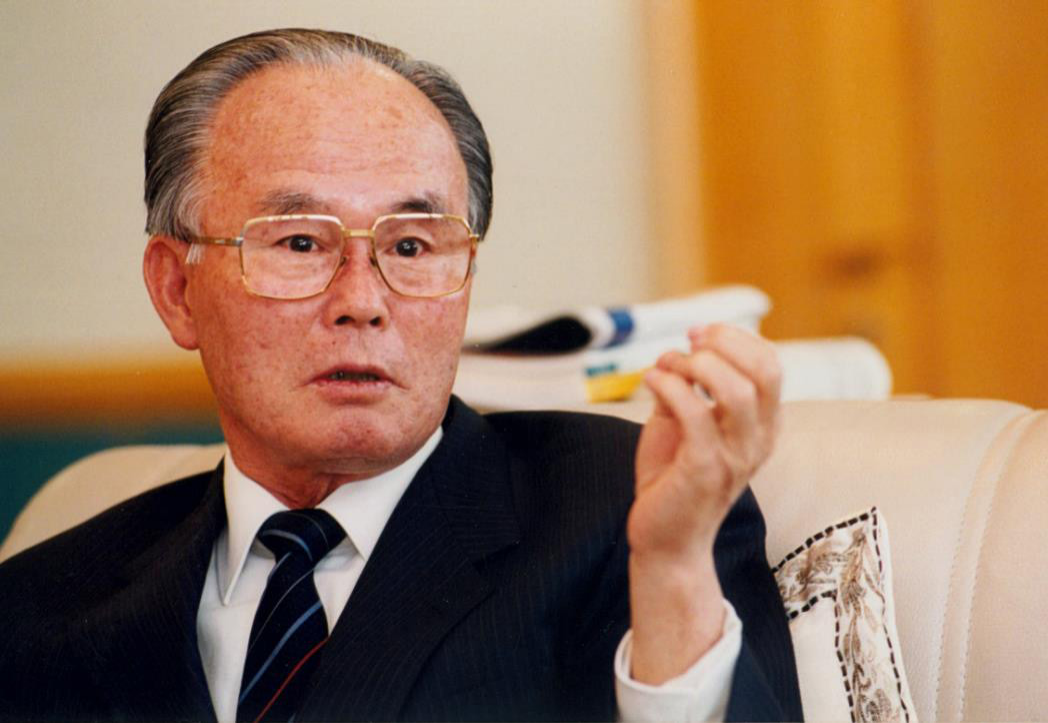
The late Dr. Yoo Bock Lee (1927–2018), Professor of Pathology, Yonsei University College of Medicine. The photo was kindly provided by his daughter, Dr. Sooyoung Lee, Professor of Pediatrics, Ajou University School of Medicine.

Dr. Yoo Bock Lee (1927–2018) formally raised the necessity of medical school accreditation in Korea in the article “Need for medical school assessment system” in the Korean Journal of Medical Education in 1990 (Fig. 1). Dr. Lee, a professor of pathology at Yonsei University, proposed 2 evaluation processes to improve medical education as follows [10]:

First, an assessment of the structure and process of medical education is needed. The fastest way to achieve this goal will be to establish an accreditation system, which will enable the standardization of the quality of medical schools. Furthermore, such a system will ensure that medical school graduates will be equipped with a certain level of knowledge, skills, and attitudes. Second, outcome assessment of graduates is also needed to provide feedback for medical education.

In November 1991, at the 22nd academic seminar held in Gwangju, Jeollanam-do (South Jeolla Province) by the KAMC, a session entitled “Medical school accreditation system” was presented. The presentations by Kim [11], Choi [12], and Kim [13] were published in the *Korean Journal of Medical Education*, volume 3, 1991. The KAMC held additional seminars in February, June, and November 1992, and continued discussions on evaluating the curriculum, professors, students, finances, and facilities. The main reason why the KAMC focused on the evaluation of medical schools was related to the issue of the possible establishment of new medical schools. The medical community in Korea, especially the KAMC, was concerned about this issue. At the brief general meeting in June 1992, the KAMC adopted a written statement that opposed establishing new medical schools, and this statement was submitted to the Ministry of Education. The written statement can be summarized as follows [14]:

Aspects of the social situation have led to calls to increase the number of physicians and facilities according to the rapid increase in demands for medical services since the 1970s. However, medical colleges also have many difficulties in properly conducting medical education. In this situation, it is unreasonable to continue establishing new medical schools. It is necessary to re-evaluate the education of existing medical schools to improve the quality of medical education. Based on the results of the evaluation, long-term medical human resources supply and demand issues, including new medical schools, can be discussed.

Consequently, this written statement spurred further discussions of the medical education accreditation system in Korea and served as a catalyst for collaboration with other medical organizations.

## History of the establishment of the Korea Institute of Medical Education and Evaluation

In the United States, where the 1st medical institution accreditation system was introduced, the Association of American Medical Colleges (AAMC) and the American Medical Association (AMA) jointly agreed to establish an independent accreditation organization, the Liaison Committee on Medical Education (LCME), in 1942. During the World War II period, the United States federal government announced plans to shorten the medical education training period and to increase the number of admissions to medical schools to address the lack of military physicians. The medical community opposed this plan because of concerns that it would cause the quality of medical education to deteriorate. This controversy led to the introduction of an accreditation system for medical schools [15]. However, in the early stage, many universities did not participate in the LCME accreditation system, which was also not mandatory. The medical education accreditation system in the United States gradually began to function properly in the mid-1970s, and other developed countries began to operate medical education accreditation systems starting in the 1980s.

Some think that the introduction of the medical education accreditation system in Korea originated from opposition to the government’s decision to allow the establishment of new medical schools in the 1980s and early 1990s. Of course, this issue sparked a debate on the necessity of introducing a medical school accreditation system. However, another fundamental reason for introducing a medical accreditation system was the medical community’s desire to promptly introduce an “autonomous” accreditation system. The Korean Council for University Education (KCUE) began to evaluate departmental units separately from universities as a whole in 1992. Departments of physics and electrical engineering were targets of evaluation in 1992. The KCUE revised the departmental evaluation plan at the government level in 1994, and suggested evaluating departments that were closely related to international competitiveness, the basic sciences, and health- and medicine-related fields. In 1995, the KCUE published “A manual for 1996 accreditation evaluation of health-related colleges” [16]. Based on this manual, the KCUE evaluated colleges of medicine, dentistry, and oriental medicine in 1996.

However, the KCUE evaluation in 1996 was a norm-referenced assessment, not a criterion-referenced assessment. Therefore, it was not sufficient to be considered a proper accreditation, which is why the medical community hastened to introduce a medical education accreditation system as a criterion-referenced assessment. The evaluation of medical schools by the KCUE in 1996 played a decisive role in introducing the autonomous medical school accreditation system.

**Fig. 2.**
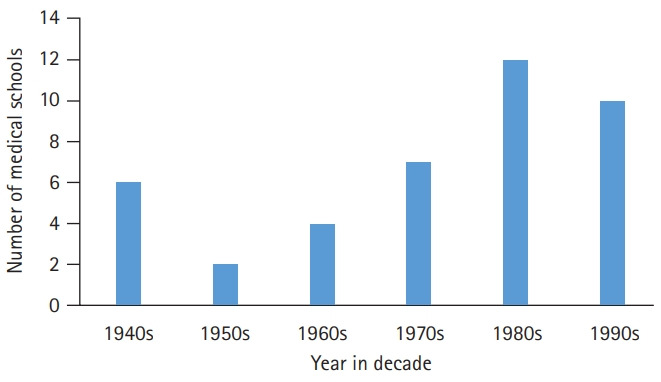
Number of newly established medical schools by decade. One school established in the 1990s was closed in 2018.

In the 1980s and 1990s, 22 medical schools were newly established (Fig. 2). The members of the KAMC strongly felt a sense of crisis regarding the quality of medical education in response to the government’s decision to allow new medical schools. The KAMC proposed organizing a council to discuss government policies on medical education and to suggest alternatives. As a result, the Korean Council of Medical Education (KCME) was created in April 1996 by representatives of 9 groups, including the Korean Hospital Association, the Korean Medical Association, the National Health Personnel Licensing Examination Board of Korea, and the KAMC.

In the meantime, the KCME commissioned Dr. Kwang-ho Meng in 1996 to conduct “A study on the development of medical school accreditation system in Korea” [17]. The KAMC and the Korean Society of Medical Education announced a voluntary medical education accreditation system at their joint conference in November 1997. The KCME established the ABMEK in July 1998, which was the predecessor organization of the KIMEE.

In 1999, the ABMEK planned a voluntary self-assessment initiative for 10 new medical schools that were excluded from the 1996 evaluation by the KCUE. However, only 8 schools participated in this self-assessment because it was not compulsory. Instead, this self-assessment was an advisory accreditation conducted in preparation for the 1st cycle of accreditation, which was scheduled to be conducted starting in 2000. The ABMEK changed its name to the KIMEE in 2003 and became an independent corporation with approval from the Ministry of Health and Welfare in 2004.

## Medical education accreditation in Korea

The important initial activities of KIMEE included preparation of standards for accreditation and an operating system, efforts for public recognition, and international exchange related to this system [18]. As mentioned above, the start of the self-assessment accreditation system in Korea’s medical schools was initially based on the medical college evaluation of the KCUE in 1996. In 1999, the ABMEK adopted the evaluation items used by KCUE, with some modifications. The number of items was 50. Out of them, 16 items were “must items,” which should be fulfilled. The 50 items were divided into 5 areas based on the education input-output model, which included the (1) educational goals and curriculum, (2) students, (3) faculty members, (4) facilities and equipment, and (5) administration and finance.

**Fig. 3.**
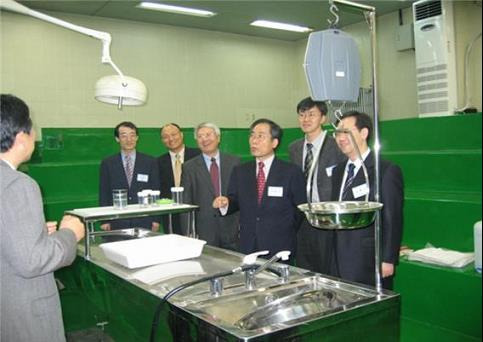
On-site visit for the 1st cycle medical school evaluation in 2000.

For the 1st cycle of medical education accreditation conducted from 2000 to 2004, the ABMEK developed a set of evaluation items, which consisted of 18 mandatory and 32 recommended items in 5 areas, as mentioned above. In this process, the ABMEK referred to the evaluation items and standards of the LCME and the Australian Medical Council, which are the medical education accreditation institutes in the United States and Australia, respectively [19,20]. Each medical school’s accreditation status was determined based on its institutional self-evaluation report and on-site observations (Fig. 3).

Following the 1st cycle of accreditation, the KIMEE completed the 2nd cycle (2007–2011) and the post-2nd cycle (2012–2018) of accreditation. Since 2019, KIMEE has been conducting the 4th cycle of accreditation using the ASK2019 (Accreditation Standards of the Korean Institute of Medical Education and Evaluation 2019), which reflect the WFME global standards. In the 4th cycle of accreditation, which is currently underway, the number of evaluation criteria (or items) has increased, and the standards have also become more rigorous in each cycle [21]. Therefore, the educational quality level and educational environment of medical schools in Korea have improved with ongoing periodic accreditation.

The establishment of legal support was another important supportive activity. Specifically, the Medical Service Act supported medical school accreditation and the Enforcement Decree of the Higher Education Act strengthened its importance, as mentioned above.

After the successful accreditation work in 2004, the KIMEE began to work towards recognition as an official accreditation body by the Korean government. Fortunately, the Ministry of Health and Welfare and the Ministry of Education, Science and Technology at the time were concerned about the accreditation system of professional fields, including medicine. Consequently, the Ministry of Education, Science, and Technology commissioned Lee [22] in 2011 to conduct a study entitled “Policy research on evaluation and accreditation of educational programs.” The National Assembly of Korea also raised the need for legislation on the medical school accreditation system by holding a public hearing [23].

Simultaneously, the KIMEE took steps to inform the international community about the accreditation activities of medical schools in Korea. In March 2003, Korean delegates attended the WFME World Congress in Copenhagen, Denmark, to introduce these autonomous medical education accreditation activities [24]. In December 2004, KIMEE members attended the 2nd Asia-Pacific Medical Education Conference in Singapore, where they publicized the contents of the accreditation system in Korea [25].

After completing the 1st cycle of accreditation, the KIMEE immediately began to prepare for the 2nd cycle of evaluation and published an English booklet entitled “The assessment and accreditation of medical education programs in Korea” in 2006 that introduced the evaluation items and standards used for the accreditation. This booklet was published and distributed to related organizations in Korea and abroad [26]. In 2016, the KIMEE was recognized as an international medical education accreditation body by the WFME.

## Achievements and meaning of early medical education accreditation activities in Korea

The 1st cycle of accreditation completed in 2004 was an opportunity to assess the adequacy and appropriateness of the criteria initially developed by the KIMEE. It also served as an excellent opportunity to confirm the responses of the schools that were evaluated [27]. The most important achievement of the 1st cycle of accreditation was that all 41 medical schools in Korea voluntarily participated in this process, although there was no legal obligation to participate.

The response from faculty members to this system was also encouraging. According to the survey results of 314 professors who actively participated in self-assessment or the local visiting committee in the 1st cycle, 80.7% said that this accreditation process improved the quality of medical education. Therefore, it was recognized as a critical system for the quality improvement of medical education [3]. The accreditation system furnished an excellent opportunity for faculty members to periodically check the overall quality of education at their medical schools, to precisely identify the strengths and weaknesses of their curriculum, and to raise the awareness of other faculty members about the importance of medical education. This system was identified as a highly effective way of obtaining support from the university administration to develop medical schools. These professors’ opinions also pointed to deficiencies in the medical curriculum and educational environment in Korea at that time. For example, when evaluating the “faculty members” area, the number of professors of basic medicine ranged from 13 to 85.

Furthermore, the number of clinical faculty members ranged from 15 to 700. In the results of the 1st cycle of accreditation, 9 medical schools were given “conditional accreditation” status; however, no public action could be taken on this result at that time. Five out of the 9 schools that received a conditional accreditation later compensated for their deficiencies and received full accreditation. It should be stressed that the results of the 1st cycle of accreditation contributed to the development of additional evaluation items or standards for the next cycle’s work and the promotion of Korea’s medical education accreditation system to the international level.

The initial activities of the KIMEE were sufficient to attract the attention of the government’s education authorities, and this 1st cycle of work ultimately confirmed the necessity of an autonomous accreditation system for professional fields.

## Conclusion

Through 3 cycles of medical school evaluation and accreditation, the Korean medical school accreditation system was well received internationally. Thus, the KIMEE did its best to improve Korea’s level of medical education through the accreditation system. Furthermore, the advancement of post-graduate training courses and graduate medical education for physicians was strengthened [28]. It is necessary to secure financial resources and to recruit experts to ensure stable operations to continue achieving these goals. However, this is difficult for the KIMEE to accomplish through only its efforts. Instead, support is needed from the medical community as a whole and related organizations. In the case of the LCME, with a history of nearly 80 years, “improving the quality of medical education” has always been the core value of the leaders of the AMA and AAMC who operate the LCME. Of course, 15 years after securing the status of an independent corporate body, the KIMEE must develop the capacity to carry out its own goals. Because medical education accreditation is a core activity for social responsibility in the field of medicine, this system has the ultimate goal of improving the quality of medical education in Korea. Therefore, the KIMEE needs to draw support and cooperation from the entire medical community.

## Figures and Tables

**Table 1.** Agencies recognized by the World Federation for Medical Education for the accreditation of medical education with their corresponding countries and recognition status

Agency	Country	Recognized until
Caribbean Accreditation Authority for Education in Medicine and Other Health Professions (CAAM-HP)	Countries of the Caribbean Community, Caribbean Community, Dominican Republic	May 2022
The Association for Evaluation and Accreditation of Medical Education Programs (TEPDAD)	Turkey, State of Palestine, Oman, Qatar, Kuwait, Lebanon	July 2023
Committee on Accreditation of Canadian Medical Schools (in cooperation with LCME)	Canada	April 2024
Liaison Committee on Medical Education (LCME)	United States of America	April 2024
Korean Institute of Medical Education and Evaluation (KIMEE)	Republic of Korea	September 2026
Accreditation Commission on Colleges of Medicine (ACCM)	Selected Caribbean countries: Anguilla, Aruba, Cayman Islands, Curacao, Dominica, Saint Kitts and Nevis, Sint Maarten, St. Vincent & the Grenadines University of Jordan Faculty of Medicine-Jordan	December 2026
Japan Accreditation Council for Medical Education (JACME)	Japan	March 2027
Australian Medical Council (AMC)	Australia and New Zealand	January 2028
Independent Agency for Accreditation and Rating (IAAR)	Kazakhstan, Kyrgyz Republic, Republic of Moldova, Russian Federation, Tajikistan	January 2028
Sudan Medical Council (SMC)	Sudan	June 2028
National Center for Educational Quality Enhancement (NCEQE)	Georgia	October 2028
Institute for Medical Education Accreditation (IMEAc)	Thailand	October 2028
Indonesian Accreditation Agency for Higher Education in Health (Lembaga Akreditasi Mandiri Perguruan Tinggi Kesehatan [IAAHEH/LAM-PTKes])	Indonesia	October 2028
Accreditation Organisation of the Netherlands and Flanders (Nederlands-Vlaamse Accreditatieorganisatie [NVAO])	Netherlands and Flanders	November 2028
Mexican Board for Accreditation of Medical Education (Consejo Mexicano para la Acreditación de la Educación Médica [COMAEM])	Mexico and Costa Rica	April 2029
National Authority for Quality Assurance and Accreditation of Education (NAQAAE)	Egypt	April 2029
System of Accreditation of Medical Schools/Sistema de Acreditação de Escolas Médicas (SAEME)	Brazil	April 2029
Taiwan Medical Accreditation Council (TMAC)	Taiwan	April 2029
Secretariat of the Council for Undergraduate Medical Education (SCUME)	Iran	June 2029
Commission for Academic Accreditation (CAA)	United Arab Emirates	June 2029
Cyprus Agency of Quality Assurance and Accreditation in Higher Education (CYQAA)	Cyprus	February 2030
Working Committee for the Accreditation of Medical Education, Ministry of Education (WCAME)	China	June 2030
Medical Council of Ireland (MCI)	Ireland Royal College of Surgeons in Ireland, Medical University of Bahrain-Bahrain	June 2030

From World Federation for Medical Education. WFME Recognition Programme [Internet]. Ferney-Voltaire (France): World Federation for Medical Education; 2020 [cited 2020 Oct 3]. Available from: https://wfme.org/accreditation/recognition-programme/ [2].

## Supplementary materials

Supplement 1. The audio recording of the abstract.
